# 16-[(*E*)-Benzyl­idene]-2-hy­droxy-12,13-diphenyl-1,11-diaza­penta­cyclo­[12.3.1.0^2,10^.0^3,8^.0^10,14^]octa­deca-3(8),4,6-triene-9,15-dione

**DOI:** 10.1107/S1600536810028345

**Published:** 2010-07-24

**Authors:** Raju Suresh Kumar, Hasnah Osman, Mohamed Ashraf Ali, Jia Hao Goh, Hoong-Kun Fun

**Affiliations:** a School of Chemical Sciences, Universiti Sains Malaysia, 11800 USM, Penang, Malaysia; b Institute for Research in Molecular Medicine, Universiti Sains Malaysia, 11800 USM, Penang, Malaysia; c X-ray Crystallography Unit, School of Physics, Universiti Sains Malaysia, 11800 USM, Penang, Malaysia

## Abstract

In the title compound, C_35_H_28_N_2_O_3_, an intra­molecular O—H⋯N hydrogen bonds generates a five-membered ring, producing an *S*(5) ring motif. The piperidone ring adopts a half-chair conformation and the two pyrrolidine rings adopt an envelope conformation. The dihedral angles formed between adjacent benzene rings are 74.39 (5) and 37.70 (6)°. In the crystal crystal, inter­molecular C—H⋯O hydrogen bonds link mol­ecules into dimers, which are further inter­connected into two-dimensional networks parallel to the *ac* plane by inter­molecular C—H⋯O hydrogen bonds. The crystal structure is consolidated by weak C—H⋯π inter­actions.

## Related literature

For general background to and applications of the title compound, see: Daly *et al.* (1986 ▶); Monlineux & Pelletier (1987 ▶); Padwa (1984 ▶); Tsuge & Kanemasa (1989 ▶); Waldmann (1995 ▶). For ring puckering analysis, see: Cremer & Pople (1975 ▶). For graph-set descriptions of hydrogen-bond ring motifs, see: Bernstein *et al.* (1995 ▶). For closely related structures, see: Kumar *et al.* (2010**a* ▶,b* ▶). For the stability of the temperature controller used in the data collection, see: Cosier & Glazer (1986 ▶).
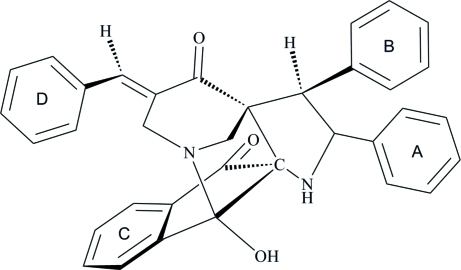


## Experimental

### 

#### Crystal data

C_35_H_28_N_2_O_3_
*M* *_r_* = 524.59
Triclinic, 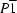

*a* = 8.6319 (2) Å
*b* = 11.8130 (2) Å
*c* = 14.3562 (3) Å
α = 75.395 (1)°
β = 72.876 (1)°
γ = 76.185 (1)°
*V* = 1332.18 (5) Å^3^
*Z* = 2
Mo *K*α radiation
μ = 0.08 mm^−1^
*T* = 100 K
0.32 × 0.30 × 0.25 mm

#### Data collection

Bruker SMART APEXII CCD area-detector diffractometer
Absorption correction: multi-scan (*SADABS*; Bruker, 2009 ▶) *T*_min_ = 0.973, *T*_max_ = 0.980
35745 measured reflections
10026 independent reflections
8085 reflections with *I* > 2σ(*I*)
*R*_int_ = 0.027

#### Refinement

*R*[*F* ^2^ > 2σ(*F* ^2^)] = 0.048
*wR*(*F* ^2^) = 0.136
*S* = 1.02
10026 reflections
369 parameters
H atoms treated by a mixture of independent and constrained refinement
Δρ_max_ = 0.51 e Å^−3^
Δρ_min_ = −0.24 e Å^−3^

### 

Data collection: *APEX2* (Bruker, 2009 ▶); cell refinement: *SAINT* (Bruker, 2009 ▶); data reduction: *SAINT*; program(s) used to solve structure: *SHELXTL* (Sheldrick, 2008 ▶); program(s) used to refine structure: *SHELXTL*; molecular graphics: *SHELXTL*; software used to prepare material for publication: *SHELXTL* and *PLATON* (Spek, 2009 ▶).

## Supplementary Material

Crystal structure: contains datablocks global, I. DOI: 10.1107/S1600536810028345/rz2477sup1.cif

Structure factors: contains datablocks I. DOI: 10.1107/S1600536810028345/rz2477Isup2.hkl

Additional supplementary materials:  crystallographic information; 3D view; checkCIF report

## Figures and Tables

**Table 1:** Hydrogen-bond geometry (Å, °) *Cg*1 and *Cg*2 are the centroids of the C30–C35 and C9–C14 benzene rings, respectively.

*D*—H⋯*A*	*D*—H	H⋯*A*	*D*⋯*A*	*D*—H⋯*A*
O1—H1*O*1⋯N1	0.862 (18)	1.999 (18)	2.6383 (12)	130.2 (15)
C7—H7*A*⋯O1^i^	0.98	2.49	3.4701 (13)	177
C10—H10*A*⋯O1^i^	0.93	2.44	3.3605 (13)	173
C35—H35*A*⋯O3^ii^	0.93	2.44	3.3424 (16)	163
C13—H13*A*⋯*Cg*1^iii^	0.93	2.89	3.7470 (12)	155
C20—H20*A*⋯*Cg*2^iv^	0.93	2.84	3.4275 (15)	122
C33—H33*A*⋯*Cg*2^v^	0.93	2.96	3.7723 (15)	147

Symmetry codes: (i) 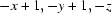; (ii) 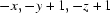; (iii) 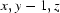; (iv) 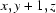; (v) 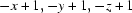.

**Fig. 1.**
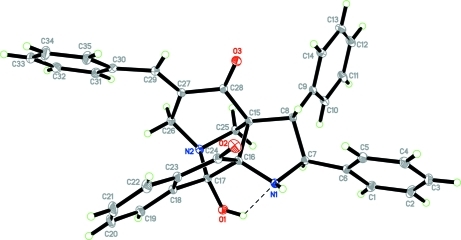
The molecular structure of the title compound, showing 30 % probability displacement ellipsoids for non-H atoms and the atom-numbering scheme. An intramolecular hydrogen bond is shown as dashed line.

**Fig. 2.**
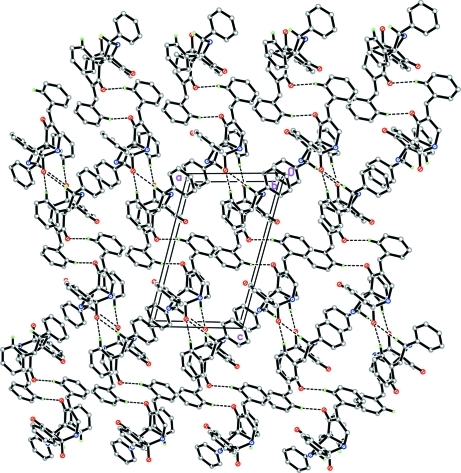
The crystal structure of the title compound, viewed down the b axis, showing a two-dimensional hydrogen-bonded network parallel to the ac plane. H atoms not involved in intermolecular hydrogen bonds (dashed lines) have been omitted for clarity.

## supplementary crystallographic information

### Comment

The 1,3-dipolar cycloaddition of azomethine ylides with olefinic dipolarophiles offers an excellent route for the construction of pyrrolidines (Tsuge & Kanemasa, 1989; Padwa, 1984). The chemistry of azomethine ylides has gained significance in recent years as it serves as an important route for the construction of nitrogen containing five-membered heterocycles, which are often central ring systems of numerous natural products (Daly *et al.*, 1986; Waldmann, 1995). The pyrrolidine moiety is one of the significant core structures among the most extensively studied natural and synthetic heterocyclic compounds with remarkable medicinal activities (Monlineux & Pelletier, 1987).

In the title compound (Fig. 1), an intramolecular O1—H1O1···N1 hydrogen bond (Table 1) forms a five-membered ring, generating an *S*(5) hydrogen bond ring motif (Bernstein *et al.*, 1995). The 4-piperidone ring (N2/C15/C25-C28) adopts a half-chair conformation, with puckering parameters Q = 0.6229 (10) Å, θ = 137.36 (9)° and φ = 236.70 (14)° (Cremer & Pople, 1975). The two fused pyrrolidine rings (N1/C7/C8/C15/C16 and N2/C15/C16/C17/C25) adopt an envelope conformation, with atoms C8 and C25 as the flap atoms, respectively. The puckering parameters are Q = 0.4071 (10) Å, φ = 248.43 (14)° for the N1/C7/C8/C15/C16 ring and Q = 0.4468 (10) Å, φ = 151.15 (13)° for the N2/C15/C16/C17/C25 pyrrolidine ring. The dihedral angles formed between benzene rings A/B and C/D are 74.39 (5) and 37.70 (6)°, respectively. The bond lengths and angles are comparable to those observed in closely related structures (Kumar *et al.*, 2010**a*,b*).

In the crystal structure (Fig. 2), intermolecular C35—H35A···O3 hydrogen bonds (Table 1) link neighbouring molecules into dimers. Intermolecular C7—H7A···O1 and C10—H10A···O1 hydrogen bonds (Table 1) further interconnect these dimers into two-dimensional networks parallel to the *ac* plane. The crystal structure is further stabilized by weak intermolecular C13—H13A···*Cg*1, C20—H20A···*Cg*2 and C33—H33A···*Cg*2 interactions, where *Cg*1 and *Cg*2 are the centroids of the C30-C35 and C9-C14 benzene rings, respectively.

### Experimental

A mixture of 3,5-bis[(*E*)-phenylmethylidene]tetrahydro-4(1*H*)- pyridinone (0.100 g, 0.363 mmol), ninhydrin (0.065 g, 0.363 mmol) and phenylglycine (0.055 g, 0.363 mmol) were dissolved in methanol (10 ml) and refluxed for 1 h. After completion of the reaction as evident from TLC, the mixture was poured into water (50 ml). The solid precipitated was filtered and washed with water to afford the product which was recrystallized from ethyl acetate to reveal the title compound as colourless crystals.

### Refinement

Atoms H1N1 and H1O1 were located from a difference Fourier map [N1—H1N1 = 0.904 (15) Å and O1—H1O1 = 0.863 (18) Å] and allowed to refine freely. The remaining H atoms were placed in their calculated positions, with C—H = 0.93–0.97 Å, and refined using a riding model, with *U*_iso_ = 1.2 *U*_eq_(C).

### Crystal data

**Table:**

C_35_H_28_N_2_O_3_	*Z* = 2
*M**_r_* = 524.59	*F*(000) = 552
Triclinic, *P*1	*D*_x_ = 1.308 Mg m^−^^3^
Hall symbol: -P 1	Mo *K*α radiation, λ = 0.71073 Å
*a* = 8.6319 (2) Å	Cell parameters from 9908 reflections
*b* = 11.8130 (2) Å	θ = 2.5–33.0°
*c* = 14.3562 (3) Å	µ = 0.08 mm^−^^1^
α = 75.395 (1)°	*T* = 100 K
β = 72.876 (1)°	Block, colourless
γ = 76.185 (1)°	0.32 × 0.30 × 0.25 mm
*V* = 1332.18 (5) Å^3^	

### Data collection

**Table:**

Bruker SMART APEXII CCD area-detector diffractometer	10026 independent reflections
Radiation source: fine-focus sealed tube	8085 reflections with *I* > 2σ(*I*)
graphite	*R*_int_ = 0.027
φ and ω scans	θ_max_ = 33.1°, θ_min_ = 1.5°
Absorption correction: multi-scan (*SADABS*; Bruker, 2009)	*h* = −13→13
*T*_min_ = 0.973, *T*_max_ = 0.980	*k* = −15→18
35745 measured reflections	*l* = −21→22

### Refinement

**Table:**

Refinement on *F*^2^	Primary atom site location: structure-invariant direct methods
Least-squares matrix: full	Secondary atom site location: difference Fourier map
*R*[*F*^2^ > 2σ(*F*^2^)] = 0.048	Hydrogen site location: inferred from neighbouring sites
*wR*(*F*^2^) = 0.136	H atoms treated by a mixture of independent and constrained refinement
*S* = 1.02	*w* = 1/[σ^2^(*F*_o_^2^) + (0.0752*P*)^2^ + 0.3278*P*] where *P* = (*F*_o_^2^ + 2*F*_c_^2^)/3
10026 reflections	(Δ/σ)_max_ < 0.001
369 parameters	Δρ_max_ = 0.51 e Å^−^^3^
0 restraints	Δρ_min_ = −0.24 e Å^−^^3^

### Special details

**Table:**

Experimental. The crystal was placed in the cold stream of an Oxford Cryosystems Cobra open-flow nitrogen cryostat (Cosier & Glazer, 1986) operating at 100.0 (1)K.
Geometry. All esds (except the esd in the dihedral angle between two l.s. planes) are estimated using the full covariance matrix. The cell esds are taken into account individually in the estimation of esds in distances, angles and torsion angles; correlations between esds in cell parameters are only used when they are defined by crystal symmetry. An approximate (isotropic) treatment of cell esds is used for estimating esds involving l.s. planes.
Refinement. Refinement of F^2^ against ALL reflections. The weighted R-factor wR and goodness of fit S are based on F^2^, conventional R-factors R are based on F, with F set to zero for negative F^2^. The threshold expression of F^2^ > 2sigma(F^2^) is used only for calculating R-factors(gt) etc. and is not relevant to the choice of reflections for refinement. R-factors based on F^2^ are statistically about twice as large as those based on F, and R- factors based on ALL data will be even larger.

### Fractional atomic coordinates and isotropic or equivalent isotropic displacement parameters (Å^2^)

**Table:**

	*x*	*y*	*z*	*U*_iso_*/*U*_eq_	
O1	0.37832 (9)	0.64683 (7)	0.04084 (5)	0.01893 (14)	
O2	−0.08312 (9)	0.57843 (7)	0.29194 (6)	0.02456 (16)	
O3	0.17358 (9)	0.37449 (7)	0.41701 (5)	0.01996 (15)	
N1	0.15205 (10)	0.51021 (7)	0.10911 (6)	0.01565 (15)	
N2	0.46901 (10)	0.57415 (7)	0.18617 (6)	0.01554 (15)	
C1	0.20254 (13)	0.27449 (9)	−0.01412 (8)	0.02146 (19)	
H1A	0.3056	0.2903	−0.0519	0.026*	
C2	0.12201 (16)	0.20652 (10)	−0.04456 (10)	0.0284 (2)	
H2A	0.1715	0.1772	−0.1027	0.034*	
C3	−0.03146 (16)	0.18240 (10)	0.01147 (10)	0.0290 (2)	
H3A	−0.0834	0.1350	−0.0080	0.035*	
C4	−0.10753 (14)	0.22921 (10)	0.09674 (9)	0.0264 (2)	
H4A	−0.2116	0.2145	0.1336	0.032*	
C5	−0.02863 (12)	0.29778 (9)	0.12704 (8)	0.02011 (18)	
H5A	−0.0808	0.3299	0.1836	0.024*	
C6	0.12871 (11)	0.31887 (8)	0.07312 (7)	0.01559 (16)	
C7	0.22380 (11)	0.38226 (8)	0.11019 (7)	0.01398 (16)	
H7A	0.3361	0.3768	0.0674	0.017*	
C8	0.23468 (11)	0.33085 (8)	0.21896 (7)	0.01388 (15)	
H8A	0.1220	0.3279	0.2598	0.017*	
C9	0.33395 (11)	0.20676 (8)	0.23764 (7)	0.01492 (16)	
C10	0.47150 (12)	0.16476 (8)	0.16708 (8)	0.01862 (18)	
H10A	0.5069	0.2147	0.1067	0.022*	
C11	0.55647 (13)	0.04853 (9)	0.18631 (9)	0.0231 (2)	
H11A	0.6488	0.0219	0.1390	0.028*	
C12	0.50438 (14)	−0.02770 (9)	0.27554 (9)	0.0245 (2)	
H12A	0.5611	−0.1052	0.2880	0.029*	
C13	0.36703 (14)	0.01263 (9)	0.34600 (8)	0.0241 (2)	
H13A	0.3310	−0.0381	0.4058	0.029*	
C14	0.28311 (13)	0.12884 (9)	0.32735 (8)	0.01999 (18)	
H14A	0.1916	0.1552	0.3753	0.024*	
C15	0.29208 (11)	0.43124 (8)	0.24213 (7)	0.01354 (15)	
C16	0.19664 (11)	0.54615 (8)	0.18681 (7)	0.01378 (15)	
C17	0.32511 (11)	0.63251 (8)	0.14526 (7)	0.01468 (16)	
C18	0.23354 (12)	0.74740 (8)	0.17864 (7)	0.01695 (17)	
C19	0.28408 (14)	0.85751 (9)	0.15035 (9)	0.0227 (2)	
H19A	0.3879	0.8663	0.1091	0.027*	
C20	0.17512 (16)	0.95369 (10)	0.18545 (10)	0.0291 (2)	
H20A	0.2058	1.0281	0.1666	0.035*	
C21	0.01984 (16)	0.94031 (11)	0.24878 (11)	0.0319 (3)	
H21A	−0.0508	1.0059	0.2718	0.038*	
C22	−0.03022 (14)	0.83114 (10)	0.27771 (9)	0.0261 (2)	
H22A	−0.1329	0.8220	0.3204	0.031*	
C23	0.07820 (12)	0.73506 (9)	0.24084 (7)	0.01799 (17)	
C24	0.04469 (11)	0.61565 (8)	0.24970 (7)	0.01689 (17)	
C25	0.47371 (11)	0.44701 (8)	0.19245 (7)	0.01582 (16)	
H25A	0.5466	0.3976	0.2328	0.019*	
H25B	0.5099	0.4272	0.1269	0.019*	
C26	0.45734 (12)	0.59095 (8)	0.28658 (7)	0.01673 (17)	
H26A	0.4352	0.6756	0.2860	0.020*	
H26B	0.5633	0.5584	0.3019	0.020*	
C27	0.32554 (11)	0.53394 (8)	0.36921 (7)	0.01594 (16)	
C28	0.25652 (11)	0.43883 (8)	0.35060 (7)	0.01509 (16)	
C29	0.25868 (13)	0.56680 (9)	0.45800 (7)	0.01835 (17)	
H29A	0.1739	0.5284	0.4999	0.022*	
C30	0.30193 (13)	0.65502 (9)	0.49733 (7)	0.01963 (18)	
C31	0.46073 (14)	0.68084 (9)	0.47512 (8)	0.0221 (2)	
H31A	0.5459	0.6442	0.4295	0.027*	
C32	0.49189 (15)	0.76115 (10)	0.52097 (9)	0.0255 (2)	
H32A	0.5978	0.7772	0.5060	0.031*	
C33	0.36614 (17)	0.81721 (10)	0.58864 (9)	0.0282 (2)	
H33A	0.3873	0.8706	0.6192	0.034*	
C34	0.20874 (18)	0.79293 (12)	0.61028 (9)	0.0321 (3)	
H34A	0.1235	0.8313	0.6548	0.039*	
C35	0.17695 (15)	0.71182 (11)	0.56616 (8)	0.0273 (2)	
H35A	0.0711	0.6951	0.5827	0.033*	
H1O1	0.306 (2)	0.6203 (15)	0.0257 (13)	0.039 (4)*	
H1N1	0.0415 (18)	0.5227 (13)	0.1190 (11)	0.023 (3)*	

### Atomic displacement parameters (Å^2^)

**Table:**

	*U*^11^	*U*^22^	*U*^33^	*U*^12^	*U*^13^	*U*^23^
O1	0.0219 (3)	0.0209 (3)	0.0135 (3)	−0.0075 (3)	−0.0018 (2)	−0.0021 (2)
O2	0.0173 (3)	0.0246 (4)	0.0276 (4)	−0.0041 (3)	0.0013 (3)	−0.0054 (3)
O3	0.0236 (3)	0.0197 (3)	0.0165 (3)	−0.0087 (3)	−0.0021 (3)	−0.0024 (3)
N1	0.0182 (3)	0.0117 (3)	0.0182 (4)	−0.0011 (3)	−0.0068 (3)	−0.0038 (3)
N2	0.0160 (3)	0.0140 (3)	0.0167 (4)	−0.0034 (3)	−0.0031 (3)	−0.0037 (3)
C1	0.0224 (4)	0.0217 (5)	0.0223 (5)	0.0003 (3)	−0.0079 (4)	−0.0090 (4)
C2	0.0363 (6)	0.0234 (5)	0.0324 (6)	0.0040 (4)	−0.0186 (5)	−0.0148 (4)
C3	0.0388 (6)	0.0173 (4)	0.0413 (7)	−0.0053 (4)	−0.0270 (5)	−0.0041 (4)
C4	0.0266 (5)	0.0228 (5)	0.0340 (6)	−0.0103 (4)	−0.0164 (4)	0.0027 (4)
C5	0.0192 (4)	0.0204 (4)	0.0220 (5)	−0.0056 (3)	−0.0070 (3)	−0.0024 (4)
C6	0.0175 (4)	0.0125 (4)	0.0176 (4)	−0.0014 (3)	−0.0065 (3)	−0.0030 (3)
C7	0.0156 (4)	0.0118 (4)	0.0147 (4)	−0.0020 (3)	−0.0036 (3)	−0.0035 (3)
C8	0.0154 (4)	0.0116 (3)	0.0144 (4)	−0.0025 (3)	−0.0030 (3)	−0.0028 (3)
C9	0.0161 (4)	0.0115 (4)	0.0182 (4)	−0.0034 (3)	−0.0056 (3)	−0.0024 (3)
C10	0.0170 (4)	0.0131 (4)	0.0237 (5)	−0.0028 (3)	−0.0027 (3)	−0.0026 (3)
C11	0.0181 (4)	0.0151 (4)	0.0338 (6)	−0.0006 (3)	−0.0044 (4)	−0.0054 (4)
C12	0.0249 (5)	0.0130 (4)	0.0367 (6)	−0.0018 (3)	−0.0139 (4)	−0.0006 (4)
C13	0.0303 (5)	0.0168 (4)	0.0248 (5)	−0.0061 (4)	−0.0111 (4)	0.0034 (4)
C14	0.0244 (4)	0.0164 (4)	0.0182 (4)	−0.0046 (3)	−0.0053 (3)	−0.0007 (3)
C15	0.0151 (4)	0.0114 (3)	0.0141 (4)	−0.0021 (3)	−0.0033 (3)	−0.0031 (3)
C16	0.0149 (4)	0.0113 (4)	0.0145 (4)	−0.0020 (3)	−0.0024 (3)	−0.0030 (3)
C17	0.0165 (4)	0.0130 (4)	0.0139 (4)	−0.0037 (3)	−0.0020 (3)	−0.0027 (3)
C18	0.0212 (4)	0.0129 (4)	0.0184 (4)	−0.0027 (3)	−0.0076 (3)	−0.0031 (3)
C19	0.0280 (5)	0.0151 (4)	0.0281 (5)	−0.0056 (4)	−0.0122 (4)	−0.0023 (4)
C20	0.0376 (6)	0.0141 (4)	0.0421 (7)	−0.0027 (4)	−0.0198 (5)	−0.0074 (4)
C21	0.0360 (6)	0.0198 (5)	0.0449 (7)	0.0053 (4)	−0.0162 (5)	−0.0178 (5)
C22	0.0247 (5)	0.0238 (5)	0.0315 (6)	0.0038 (4)	−0.0078 (4)	−0.0149 (4)
C23	0.0203 (4)	0.0151 (4)	0.0196 (4)	−0.0001 (3)	−0.0061 (3)	−0.0067 (3)
C24	0.0169 (4)	0.0158 (4)	0.0167 (4)	−0.0004 (3)	−0.0035 (3)	−0.0040 (3)
C25	0.0145 (4)	0.0138 (4)	0.0186 (4)	−0.0019 (3)	−0.0024 (3)	−0.0047 (3)
C26	0.0170 (4)	0.0164 (4)	0.0181 (4)	−0.0048 (3)	−0.0048 (3)	−0.0036 (3)
C27	0.0177 (4)	0.0150 (4)	0.0163 (4)	−0.0036 (3)	−0.0057 (3)	−0.0027 (3)
C28	0.0161 (4)	0.0137 (4)	0.0157 (4)	−0.0020 (3)	−0.0042 (3)	−0.0037 (3)
C29	0.0236 (4)	0.0166 (4)	0.0162 (4)	−0.0060 (3)	−0.0053 (3)	−0.0030 (3)
C30	0.0279 (5)	0.0175 (4)	0.0154 (4)	−0.0071 (3)	−0.0066 (3)	−0.0024 (3)
C31	0.0258 (5)	0.0205 (4)	0.0238 (5)	−0.0030 (4)	−0.0134 (4)	−0.0033 (4)
C32	0.0321 (5)	0.0220 (5)	0.0288 (5)	−0.0077 (4)	−0.0184 (4)	−0.0005 (4)
C33	0.0472 (7)	0.0222 (5)	0.0221 (5)	−0.0146 (5)	−0.0132 (5)	−0.0032 (4)
C34	0.0446 (7)	0.0310 (6)	0.0240 (5)	−0.0169 (5)	0.0017 (5)	−0.0137 (5)
C35	0.0323 (5)	0.0302 (6)	0.0218 (5)	−0.0150 (4)	0.0031 (4)	−0.0121 (4)

### Geometric parameters (Å, °)

**Table:**

O1—C17	1.4109 (11)	C15—C28	1.5175 (13)
O1—H1O1	0.863 (18)	C15—C25	1.5542 (13)
O2—C24	1.2171 (12)	C15—C16	1.5683 (12)
O3—C28	1.2214 (11)	C16—C24	1.5401 (13)
N1—C16	1.4636 (12)	C16—C17	1.5717 (13)
N1—C7	1.4883 (12)	C17—C18	1.5129 (13)
N1—H1N1	0.904 (15)	C18—C23	1.3926 (14)
N2—C25	1.4731 (12)	C18—C19	1.3955 (14)
N2—C26	1.4754 (13)	C19—C20	1.3887 (16)
N2—C17	1.4763 (12)	C19—H19A	0.9300
C1—C2	1.3933 (16)	C20—C21	1.3998 (19)
C1—C6	1.3969 (14)	C20—H20A	0.9300
C1—H1A	0.9300	C21—C22	1.3829 (17)
C2—C3	1.3857 (19)	C21—H21A	0.9300
C2—H2A	0.9300	C22—C23	1.3958 (14)
C3—C4	1.3892 (19)	C22—H22A	0.9300
C3—H3A	0.9300	C23—C24	1.4738 (14)
C4—C5	1.3874 (15)	C25—H25A	0.9700
C4—H4A	0.9300	C25—H25B	0.9700
C5—C6	1.3968 (14)	C26—C27	1.5242 (13)
C5—H5A	0.9300	C26—H26A	0.9700
C6—C7	1.5064 (13)	C26—H26B	0.9700
C7—C8	1.5482 (13)	C27—C29	1.3483 (14)
C7—H7A	0.9800	C27—C28	1.5012 (13)
C8—C9	1.5140 (12)	C29—C30	1.4666 (14)
C8—C15	1.5295 (13)	C29—H29A	0.9300
C8—H8A	0.9800	C30—C35	1.3970 (15)
C9—C10	1.3949 (13)	C30—C31	1.4025 (15)
C9—C14	1.4002 (13)	C31—C32	1.3953 (15)
C10—C11	1.3952 (13)	C31—H31A	0.9300
C10—H10A	0.9300	C32—C33	1.3859 (18)
C11—C12	1.3880 (16)	C32—H32A	0.9300
C11—H11A	0.9300	C33—C34	1.3847 (18)
C12—C13	1.3867 (17)	C33—H33A	0.9300
C12—H12A	0.9300	C34—C35	1.3886 (16)
C13—C14	1.3911 (14)	C34—H34A	0.9300
C13—H13A	0.9300	C35—H35A	0.9300
C14—H14A	0.9300		
			
C17—O1—H1O1	103.4 (11)	O1—C17—N2	107.75 (7)
C16—N1—C7	108.20 (7)	O1—C17—C18	112.40 (8)
C16—N1—H1N1	110.8 (9)	N2—C17—C18	115.09 (8)
C7—N1—H1N1	110.8 (9)	O1—C17—C16	109.81 (7)
C25—N2—C26	107.71 (7)	N2—C17—C16	106.84 (7)
C25—N2—C17	102.98 (7)	C18—C17—C16	104.70 (7)
C26—N2—C17	115.82 (7)	C23—C18—C19	120.49 (9)
C2—C1—C6	120.09 (10)	C23—C18—C17	111.55 (8)
C2—C1—H1A	120.0	C19—C18—C17	127.87 (9)
C6—C1—H1A	120.0	C20—C19—C18	118.18 (11)
C3—C2—C1	120.29 (11)	C20—C19—H19A	120.9
C3—C2—H2A	119.9	C18—C19—H19A	120.9
C1—C2—H2A	119.9	C19—C20—C21	120.99 (11)
C2—C3—C4	119.84 (10)	C19—C20—H20A	119.5
C2—C3—H3A	120.1	C21—C20—H20A	119.5
C4—C3—H3A	120.1	C22—C21—C20	121.03 (10)
C5—C4—C3	120.16 (11)	C22—C21—H21A	119.5
C5—C4—H4A	119.9	C20—C21—H21A	119.5
C3—C4—H4A	119.9	C21—C22—C23	117.91 (11)
C4—C5—C6	120.41 (10)	C21—C22—H22A	121.0
C4—C5—H5A	119.8	C23—C22—H22A	121.0
C6—C5—H5A	119.8	C18—C23—C22	121.38 (10)
C5—C6—C1	119.14 (9)	C18—C23—C24	110.55 (8)
C5—C6—C7	121.33 (9)	C22—C23—C24	127.71 (10)
C1—C6—C7	119.45 (9)	O2—C24—C23	127.65 (9)
N1—C7—C6	113.67 (7)	O2—C24—C16	124.17 (9)
N1—C7—C8	104.17 (7)	C23—C24—C16	107.95 (8)
C6—C7—C8	114.21 (7)	N2—C25—C15	103.48 (7)
N1—C7—H7A	108.2	N2—C25—H25A	111.1
C6—C7—H7A	108.2	C15—C25—H25A	111.1
C8—C7—H7A	108.2	N2—C25—H25B	111.1
C9—C8—C15	117.66 (7)	C15—C25—H25B	111.1
C9—C8—C7	115.68 (7)	H25A—C25—H25B	109.0
C15—C8—C7	101.04 (7)	N2—C26—C27	114.98 (8)
C9—C8—H8A	107.3	N2—C26—H26A	108.5
C15—C8—H8A	107.3	C27—C26—H26A	108.5
C7—C8—H8A	107.3	N2—C26—H26B	108.5
C10—C9—C14	118.20 (9)	C27—C26—H26B	108.5
C10—C9—C8	122.50 (8)	H26A—C26—H26B	107.5
C14—C9—C8	119.25 (8)	C29—C27—C28	116.36 (9)
C9—C10—C11	120.58 (9)	C29—C27—C26	124.55 (9)
C9—C10—H10A	119.7	C28—C27—C26	119.00 (8)
C11—C10—H10A	119.7	O3—C28—C27	122.90 (9)
C12—C11—C10	120.53 (10)	O3—C28—C15	122.12 (9)
C12—C11—H11A	119.7	C27—C28—C15	114.94 (8)
C10—C11—H11A	119.7	C27—C29—C30	128.62 (9)
C13—C12—C11	119.47 (9)	C27—C29—H29A	115.7
C13—C12—H12A	120.3	C30—C29—H29A	115.7
C11—C12—H12A	120.3	C35—C30—C31	118.20 (10)
C12—C13—C14	120.07 (10)	C35—C30—C29	117.01 (9)
C12—C13—H13A	120.0	C31—C30—C29	124.68 (10)
C14—C13—H13A	120.0	C32—C31—C30	120.50 (10)
C13—C14—C9	121.14 (10)	C32—C31—H31A	119.8
C13—C14—H14A	119.4	C30—C31—H31A	119.8
C9—C14—H14A	119.4	C33—C32—C31	120.49 (11)
C28—C15—C8	117.35 (7)	C33—C32—H32A	119.8
C28—C15—C25	107.65 (7)	C31—C32—H32A	119.8
C8—C15—C25	116.98 (7)	C34—C33—C32	119.32 (10)
C28—C15—C16	108.88 (7)	C34—C33—H33A	120.3
C8—C15—C16	103.12 (7)	C32—C33—H33A	120.3
C25—C15—C16	101.25 (7)	C33—C34—C35	120.63 (11)
N1—C16—C24	110.58 (7)	C33—C34—H34A	119.7
N1—C16—C15	106.43 (7)	C35—C34—H34A	119.7
C24—C16—C15	117.72 (7)	C34—C35—C30	120.84 (11)
N1—C16—C17	113.59 (7)	C34—C35—H35A	119.6
C24—C16—C17	104.93 (7)	C30—C35—H35A	119.6
C15—C16—C17	103.62 (7)		
			
C6—C1—C2—C3	−0.08 (16)	C24—C16—C17—C18	−5.11 (9)
C1—C2—C3—C4	1.96 (17)	C15—C16—C17—C18	−129.17 (7)
C2—C3—C4—C5	−1.43 (16)	O1—C17—C18—C23	124.98 (9)
C3—C4—C5—C6	−0.98 (16)	N2—C17—C18—C23	−111.16 (9)
C4—C5—C6—C1	2.83 (14)	C16—C17—C18—C23	5.82 (10)
C4—C5—C6—C7	−173.65 (9)	O1—C17—C18—C19	−51.54 (13)
C2—C1—C6—C5	−2.30 (15)	N2—C17—C18—C19	72.33 (13)
C2—C1—C6—C7	174.25 (9)	C16—C17—C18—C19	−170.69 (10)
C16—N1—C7—C6	152.42 (8)	C23—C18—C19—C20	−0.35 (15)
C16—N1—C7—C8	27.49 (9)	C17—C18—C19—C20	175.89 (10)
C5—C6—C7—N1	−68.57 (11)	C18—C19—C20—C21	1.03 (17)
C1—C6—C7—N1	114.96 (10)	C19—C20—C21—C22	−0.48 (19)
C5—C6—C7—C8	50.78 (11)	C20—C21—C22—C23	−0.74 (18)
C1—C6—C7—C8	−125.69 (9)	C19—C18—C23—C22	−0.89 (15)
N1—C7—C8—C9	−168.61 (7)	C17—C18—C23—C22	−177.70 (9)
C6—C7—C8—C9	66.82 (10)	C19—C18—C23—C24	172.68 (9)
N1—C7—C8—C15	−40.39 (8)	C17—C18—C23—C24	−4.13 (11)
C6—C7—C8—C15	−164.96 (7)	C21—C22—C23—C18	1.42 (17)
C15—C8—C9—C10	−86.52 (11)	C21—C22—C23—C24	−170.96 (11)
C7—C8—C9—C10	32.95 (12)	C18—C23—C24—O2	−174.10 (10)
C15—C8—C9—C14	96.07 (11)	C22—C23—C24—O2	−1.04 (18)
C7—C8—C9—C14	−144.46 (9)	C18—C23—C24—C16	0.55 (11)
C14—C9—C10—C11	−0.64 (15)	C22—C23—C24—C16	173.61 (10)
C8—C9—C10—C11	−178.07 (9)	N1—C16—C24—O2	55.00 (12)
C9—C10—C11—C12	0.76 (16)	C15—C16—C24—O2	−67.59 (13)
C10—C11—C12—C13	−0.23 (17)	C17—C16—C24—O2	177.86 (9)
C11—C12—C13—C14	−0.40 (17)	N1—C16—C24—C23	−119.88 (8)
C12—C13—C14—C9	0.51 (17)	C15—C16—C24—C23	117.52 (9)
C10—C9—C14—C13	0.01 (15)	C17—C16—C24—C23	2.98 (10)
C8—C9—C14—C13	177.53 (9)	C26—N2—C25—C15	75.74 (9)
C9—C8—C15—C28	−75.97 (10)	C17—N2—C25—C15	−47.14 (9)
C7—C8—C15—C28	157.10 (7)	C28—C15—C25—N2	−72.26 (9)
C9—C8—C15—C25	54.29 (11)	C8—C15—C25—N2	153.07 (8)
C7—C8—C15—C25	−72.63 (9)	C16—C15—C25—N2	41.91 (9)
C9—C8—C15—C16	164.38 (7)	C25—N2—C26—C27	−48.63 (10)
C7—C8—C15—C16	37.46 (8)	C17—N2—C26—C27	65.98 (10)
C7—N1—C16—C24	−132.42 (8)	N2—C26—C27—C29	−158.49 (9)
C7—N1—C16—C15	−3.45 (9)	N2—C26—C27—C28	18.05 (12)
C7—N1—C16—C17	109.92 (8)	C29—C27—C28—O3	−16.55 (14)
C28—C15—C16—N1	−147.40 (7)	C26—C27—C28—O3	166.63 (9)
C8—C15—C16—N1	−22.08 (9)	C29—C27—C28—C15	161.28 (8)
C25—C15—C16—N1	99.34 (8)	C26—C27—C28—C15	−15.54 (12)
C28—C15—C16—C24	−22.72 (11)	C8—C15—C28—O3	−6.06 (13)
C8—C15—C16—C24	102.61 (9)	C25—C15—C28—O3	−140.53 (9)
C25—C15—C16—C24	−135.97 (8)	C16—C15—C28—O3	110.49 (10)
C28—C15—C16—C17	92.55 (8)	C8—C15—C28—C27	176.10 (7)
C8—C15—C16—C17	−142.13 (7)	C25—C15—C28—C27	41.63 (10)
C25—C15—C16—C17	−20.70 (8)	C16—C15—C28—C27	−67.36 (9)
C25—N2—C17—O1	−84.81 (8)	C28—C27—C29—C30	178.98 (9)
C26—N2—C17—O1	157.91 (7)	C26—C27—C29—C30	−4.39 (16)
C25—N2—C17—C18	148.90 (8)	C27—C29—C30—C35	151.84 (11)
C26—N2—C17—C18	31.62 (11)	C27—C29—C30—C31	−32.02 (17)
C25—N2—C17—C16	33.14 (9)	C35—C30—C31—C32	0.05 (15)
C26—N2—C17—C16	−84.14 (9)	C29—C30—C31—C32	−176.05 (9)
N1—C16—C17—O1	−5.11 (10)	C30—C31—C32—C33	−0.44 (16)
C24—C16—C17—O1	−126.00 (8)	C31—C32—C33—C34	−0.13 (17)
C15—C16—C17—O1	109.94 (8)	C32—C33—C34—C35	1.09 (19)
N1—C16—C17—N2	−121.70 (8)	C33—C34—C35—C30	−1.5 (2)
C24—C16—C17—N2	117.40 (8)	C31—C30—C35—C34	0.91 (17)
C15—C16—C17—N2	−6.65 (9)	C29—C30—C35—C34	177.31 (11)
N1—C16—C17—C18	115.78 (8)		

### Hydrogen-bond geometry (Å, °)

**Table:**

Cg1 and Cg2 are the centroids of the C30–C35 and C9–C14 benzene rings, respectively.

**Table:**

*D*—H···*A*	*D*—H	H···*A*	*D*···*A*	*D*—H···*A*
O1—H1O1···N1	0.862 (18)	1.999 (18)	2.6383 (12)	130.2 (15)
C7—H7A···O1^i^	0.98	2.49	3.4701 (13)	177
C10—H10A···O1^i^	0.93	2.44	3.3605 (13)	173
C35—H35A···O3^ii^	0.93	2.44	3.3424 (16)	163
C13—H13A···Cg1^iii^	0.93	2.89	3.7470 (12)	155
C20—H20A···Cg2^iv^	0.93	2.84	3.4275 (15)	122
C33—H33A···Cg2^v^	0.93	2.96	3.7723 (15)	147

Symmetry codes: (i) −*x*+1, −*y*+1, −*z*; (ii) −*x*, −*y*+1, −*z*+1; (iii) *x*, *y*−1, *z*; (iv) *x*, *y*+1, *z*; (v) −*x*+1, −*y*+1, −*z*+1.
